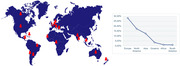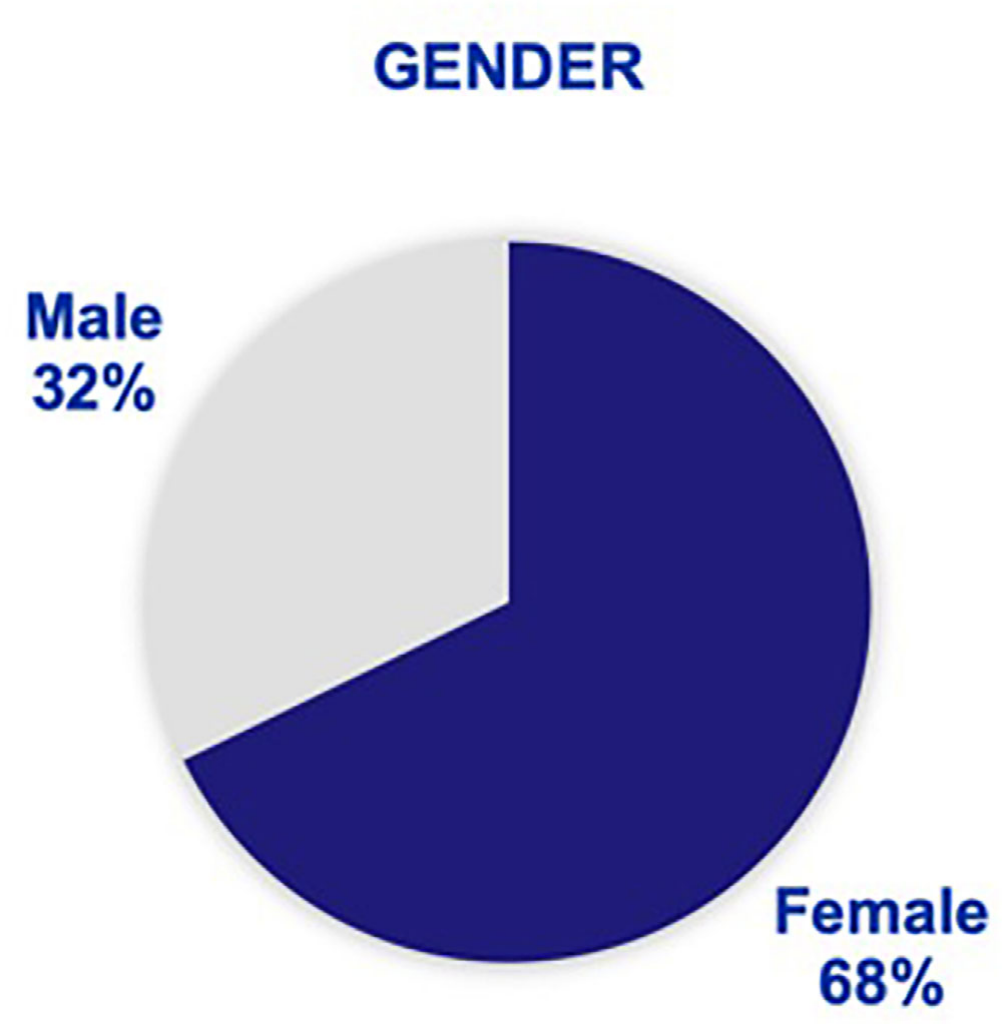# Word Young Leaders of Dementia Roadmap 2024‐2029: Approaches and Global Goals for Dementia Challenges

**DOI:** 10.1002/alz.095373

**Published:** 2025-01-09

**Authors:** Maria Florencia Clarens, Micaela Maria Arruabarrena, Claudia Kimie Suemoto, Jorge J. Llibre‐Guerra, Lucía Crivelli, Claire Sexton, Ozama Ismail

**Affiliations:** ^1^ Fleni, Buenos Aires, CABA Argentina; ^2^ Division of Geriatrics, University of São Paulo Medical School, São Paulo, São Paulo Brazil; ^3^ Department of Neurology, Washington University School of Medicine, St Louis, MO USA; ^4^ Alzheimer’s Association, Chicago, IL USA

## Abstract

**Background:**

The 2021 WHO report highlights the importance of global planning for dementia. Initiatives that unite and support young leaders and stakeholders from diverse backgrounds in dementia care & research are crucial. The G8 Summit on Dementia in 2013 led to the creation of the World Dementia Council and the World Young Leaders in Dementia (WYLD) network, which connects professionals to work together on finding solutions to dementia challenges. With the emergence of new leadership and structure in January 2024 we aim to present our 2024‐2029 roadmap.

**Method:**

We conducted a systematic review of the WYLD’s membership database to map out the geographical and sociodemographic landscape of our members and to further improve our recruitment of young leaders. Approaches and Goals for WYLD 2024‐2029 have been established with the advisory council and global partners, ensuring that the roadmap’s objectives are informed by a wide spectrum of insights and expert contributions.

**Result:**

Our review of current membership has revealed a diverse member network in 21 countries‐Fig. 1‐ with a majority of female professionals (67.78%)‐Fig. 2‐.

Our mission, empowering emerging leaders through innovation, collaboration, and creative problem‐solving in dementia care strategies guided us in our roadmap, influencing our strategic proposal and aims.

**WYLD Structure**: We are creating an advisory council and global partnerships by fostering strategic partnerships with leading organizations in the field. Appointments of Regional Leads in Africa, Asia, Australia/New Zealand, Europe, North America, and South America demonstrate our commitment to diversity.

Leadership Development Activities: WYLD will offer leadership development through diverse mentorship training programs, curated webinars for young members in dementia care & research, and grant opportunities for rising Young Leaders

**Outreach**: Regular and extensive distribution of young dementia leader’s work to inspire and facilitate worldwide progress in the field.

**Conclusion:**

The WYLD 2024‐2029 roadmap is a strategic blueprint that guides our mission to empower young leaders and innovate dementia care globally. Our growing, diverse membership, and collaborative initiatives set the stage for impactful change, ensuring that the vital work of these leaders is recognized and disseminated worldwide.